# Oral Gingival Cell Cigarette Smoke Exposure Induces Muscle Cell Metabolic Disruption

**DOI:** 10.1155/2016/2763160

**Published:** 2016-02-29

**Authors:** Andrea C. Baeder, Kiran Napa, Sarah T. Richardson, Oliver J. Taylor, Samantha G. Andersen, Shalene H. Wilcox, Duane R. Winden, Paul R. Reynolds, Benjamin T. Bikman

**Affiliations:** ^1^College of Dental Medicine, Roseman University of Health Sciences, South Jordan, UT 84095, USA; ^2^Department of Physiology and Developmental Biology, Brigham Young University, Provo, UT 84602, USA

## Abstract

Cigarette smoke exposure compromises health through damaging multiple physiological systems, including disrupting metabolic function. The purpose of this study was to determine the role of oral gingiva in mediating the deleterious metabolic effects of cigarette smoke exposure on skeletal muscle metabolic function. Using an in vitro conditioned medium cell model, skeletal muscle cells were incubated with medium from gingival cells treated with normal medium or medium containing suspended cigarette smoke extract (CSE). Following incubation of muscle cells with gingival cell conditioned medium, muscle cell mitochondrial respiration and insulin signaling and action were determined as an indication of overall muscle metabolic health. Skeletal muscle cells incubated with conditioned medium of CSE-treated gingival cells had a profound reduction in mitochondrial respiration and respiratory control. Furthermore, skeletal muscle cells had a greatly reduced response in insulin-stimulated Akt phosphorylation and glycogen synthesis. Altogether, these results provide a novel perspective on the mechanism whereby cigarette smoke affects systemic metabolic function. In conclusion, we found that oral gingival cells treated with CSE create an altered milieu that is sufficient to both disrupted skeletal muscle cell mitochondrial function and insulin sensitivity.

## 1. Introduction

Tobacco use is the leading cause of preventable disease in the United States [1], leading to pathologies of nearly every organ in the body, including the oral cavity [2]. Unsurprisingly, the airway and lungs carry much of the disease burden with smoke exposure [3, 4]. However, smoke exposure similarly affects systemic tissues, including the liver [5], pancreas [6], and skeletal muscle [7]. Each of these tissues is highly relevant in maintaining healthy nutrient metabolism throughout the body, including insulin sensitivity and mitochondrial function, which highlights the remarkable and deleterious impact of smoke exposure on metabolic function.

Like cigarette smoke exposure, insulin resistance, which affects roughly half of all American adults [8], has a hand in the etiology of many chronic diseases, such as atherosclerosis [9], diabetes [10], steatohepatitis [11], and more. Interestingly, cigarette smoke exposure is similarly linked with these diseases [12–14]. Indeed, research over the past two decades has established that smoke exposure is causally connected to insulin resistance in multiple models [15–17].

In addition to its effects on insulin signaling, cigarette smoke exposure also detrimentally disrupts mitochondrial function. Gannon et al. [18] found significant decay of mitochondrial function within granulosa cells of mice exposed to cigarette smoke. Additionally, we have observed a profound loss of healthy cardiomyocyte mitochondrial function in cell and animal models of cigarette smoke exposure [19].

Taken together, ample evidence suggests that cigarette smoke exposure harms metabolic function by, at a minimum, compromising insulin action and mitochondrial physiology. Nevertheless, despite these known associations, the precise process whereby airway smoke exposure links to systemic tissues like skeletal muscle is vague. Although we have recently found that alveolar type II cells are relevant cellular players in transmitting the airway smoke exposure insult to distal skeletal muscle [7], no work has explored whether the oral cavity itself is relevant in potentiating this effect in muscle. Thus, the purpose of this study was to determine the role of oral gingiva in mediating the deleterious metabolic effects of cigarette smoke exposure on skeletal muscle and metabolic function determined by muscle cell mitochondrial respiration and insulin signaling.

## 2. Materials and Methods

### 2.1. Cell Culture

Oral gingival Ca9-22 cells and C2C12 murine myoblasts were maintained in DMEM plus 10% fetal bovine serum (Invitrogen). For differentiation into myotubes, C2C12 myoblasts were grown to confluency and the medium was replaced with DMEM plus 10% horse serum (Invitrogen). Myotubes were used for experiments on day 4 of differentiation.

Cigarette smoke extract (CSE) was generated as previously described with slight modifications [7]. Briefly, one 2RF4 research cigarette (University of Kentucky, Lexington, KY) was continuously smoked by connecting the filtered end of the cigarette to a vacuum pump, pulling the particles into 5 mL of DMEM/F12 and the resulting medium was defined as 100% CSE and diluted with culture medium to 10%. The total particulate matter content of 2RF4 cigarettes is 11.7 mg/cigarette, tar is 9.7 mg/cigarette, and nicotine is 0.85 mg/cigarette.

For conditioned medium experiments, Ca9-22 cells were incubated with CSE for 4 h, washed with warmed growth medium, and then fed fresh medium for an additional 4 h. Following this fresh medium incubation, the medium was transferred to myotubes for 12 h, after which analyses were performed with the myotubes. This system ensured that CSE was not present in the myotube culture medium.

### 2.2. Protein Quantification and Quantitative Real-Time PCR

Proteins were quantified as described previously [20]. The following antibodies were used: Akt (9272) and pAkt-ser473 (9271).

### 2.3. Mitochondrial Respiration Protocol

Cells were prepared for the mitochondrial respiration assay as described previously [20]. Briefly, high-resolution O_2_ consumption was determined at 37°C in permeabilized cells using the Oroboros O_2_K Oxygraph (Innsbruck, Austria) with MiR05 respiration buffer as described previously [20, 21]. Respiration was determined by the following substrate-uncoupler-inhibitor-titration protocol [22]: electron flow through complex I was supported by glutamate + malate (10 and 2 mM, resp.) to determine oxygen consumption from proton leak (GM_*L*_). Following stabilization, ADP (2.5 mM) was added to determine oxidative phosphorylation capacity (GM_*P*_). Outer mitochondrial membrane integrity was tested by adding cytochrome* c* (10 *μ*M; not shown). Succinate was added (GMS_*P*_) for complex I + II electron flow into the Q-junction. To determine full electron transport system (ETS) capacity over oxidative phosphorylation in cells, the chemical uncoupler carbonyl cyanide 4-(trifluoromethoxy) phenylhydrazone (FCCP) was added (0.05 *μ*M, followed by 0.025 *μ*M steps until maximal O_2_ flux was reached). Lastly, residual oxygen consumption was measured by adding antimycin A (2.5 *μ*M) to block complex III action, effectively stopping any electron flow. This value provides a rate of respiration that is used as a baseline. Following respiration protocol (outlined below), samples were removed from the chambers and used for further analysis, including protein quantification.

### 2.4. Glycogen Assay

Glycogen was measured from cells indicated according to the manufacturer's instructions (BioVision Inc.; Milpitas, CA).

### 2.5. H_2_O_2_ Emission

H_2_O_2_ emission was measured using an Amplex Red Hydrogen Peroxide/Peroxidase Assay Kit (Molecular Probes; A22188). A reaction mixture containing 50 *μ*M Amplex Red and 0.1 U/mL HRP in Krebs-Ringer phosphate glucose (KRPG) buffer was prepared (145 mM NaCl, 5.7 mM sodium phosphate, 4.86 mM KCl, 0.54 mM CaCl_2_, 1.22 mM MgSO_4_, 5.5 mM glucose). The reaction mixture was prewarmed in a 96-well plate with 100 *μ*L of mixture per well. 20 *μ*L of cells suspended in KRPG buffer (~1.5 × 10^4^) was added to each well. Samples were incubated for 1 h. Fluorescence was measured with a microplate reader (Molecular Devices; Gemini EM).

### 2.6. ELISA for TNF*α*

Culture medium was collected after incubation with control or CSE-containing medium and centrifuged at 2,500 rpm for 5 min. TNF*α* was determined by sandwich ELISA according to the manufacturer's instructions (Abcam).

### 2.7. Statistics

Data are presented as the mean ± SEM. Data were compared by ANOVA with Tukey's post hoc analysis (Graphpad Prism; La Jolla, CA). Significance was set at *P* < 0.05.

## 3. Results

### 3.1. Cigarette Smoke Extract Alters Mitochondrial Function in Gingival Culture Medium-Treated Myotubes

**Figure 1:**
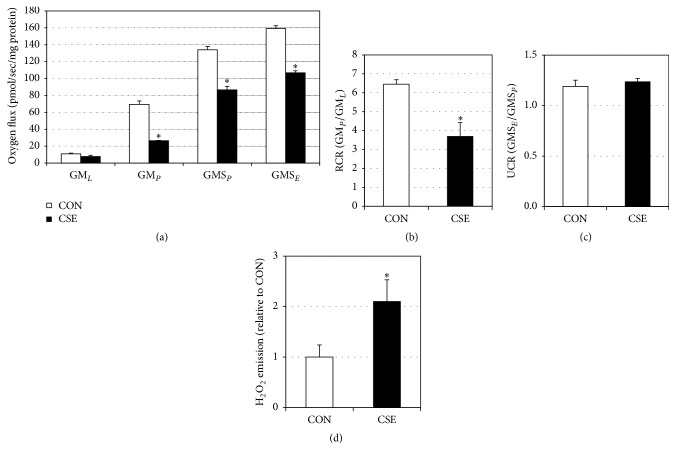
CSE-treated gingival cell conditioned medium alters myotube mitochondrial function. Myotubes were treated with conditioned medium (12 h) from gingival cells that had previously been treated with normal medium (CON) or cigarette smoke extract (CSE). To measure mitochondrial respiration ((a); *n* = 6), cells were treated with GM_*L*_: glutamate (10 mM) + malate (2 mM); GM_*D*_: + ADP (2.5 mM); GMS_*D*_: + succinate (10 mM); GMS_*F*_: + FCCP (0.05 *μ*M). Respiratory control ratio (RCR; (b)) and uncoupling control ratio (UCR; (c)) were determined by the analysis indicated. In separate experiments, myotubes were incubated with MitoTracker and imaged for analysis of H_2_O_2_ production ((d); *n* = 5). ^*∗*^ *P* < 0.05: CSE versus CON.

Our initial observations were a profound mitochondrial disruption in myotubes treated with CSE-conditioned gingival cell medium. In particular, we noted reduced oxygen consumption in the myotubes incubated with the conditioned medium from CSE-treated cells. While respiration rates were similar between conditions in the leak state (GM_*L*_) with glutamate and malate (GM), the disparity became obvious with the addition of ADP (GM_*P*_) and continued throughout the addition of succinate (GMS_*P*_) and uncoupling with FCCP (GMS_*E*_; Figure 1(a)). When comparing post hoc analysis of mitochondrial respiration, we found that myotubes treated with conditioned medium from CSE-treated gingival cells experienced a significant reduction in the respiratory control ratio (Figure 1(b)), a rough indication of mitochondrial health. However, the uncoupling control ratio (Figure 1(c)) was similar. Lastly, H_2_O_2_ production from CSE-conditioned medium-treated myotubes was significantly higher than control (Figure 1(d)), indicating increased oxidative stress.

### 3.2. Myotubes Treated with Conditioned Medium from CSE-Treated Gingival Cells Are Insulin Resistant

**Figure 2:**
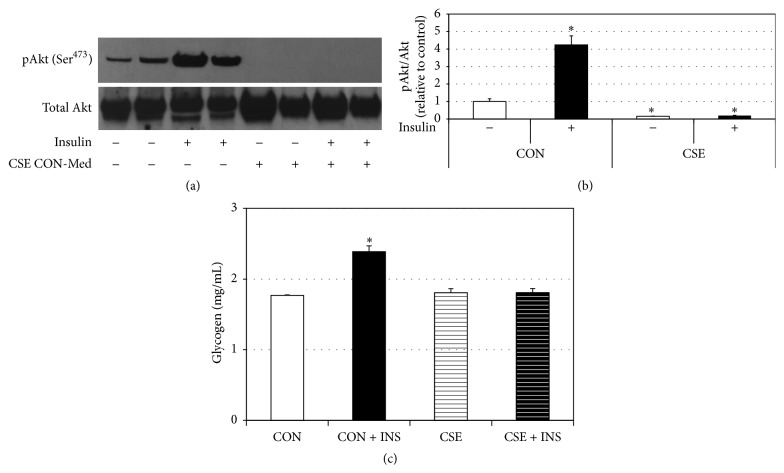
CSE-treated gingival cell conditioned medium alters myotube insulin signaling. Following treatment with conditioned medium, myotubes were stimulated with insulin (100 nM) for 10 min prior to lysing. Western blot for pAkt and total Akt ((a); *n* = 3) was performed and quantified ((b); *n* = 3). Glycogens levels were also measured in similarly treated cells ((c); *n* = 6). ^*∗*^ *P* < 0.05: CSE versus CON.

Similar to the experiments above, myotubes were treated with fresh conditioned medium from gingival cells following control (CON) or CSE treatment. Following myotube incubation with conditioned medium, myotubes were stimulated with insulin (100 nM) for 10 min. While CON cells experienced a robust increase in Akt phosphorylation, an indication of healthy insulin signaling, myotubes incubated with CSE-conditioned medium had no insulin response (Figures 2(a) and 2(b)). As further evidence, we determined myotube glycogen content, which is usually increased with insulin. Indeed, CON cells had significantly higher glycogen levels following insulin stimulation. In contrast, CSE-conditioned medium from gingival cells blocked this effect (Figure 2(c)).

### 3.3. Cigarette Smoke Extract Increases TNF*α* Secretion from Gingival Cells

**Figure 3:**
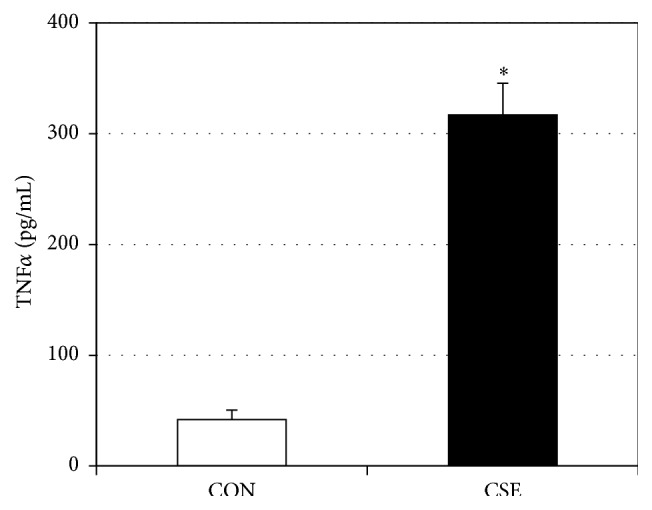
CSE-treated gingival cells release TNF*α*. Ca9-22 gingival cells were treated with control (CON) or CSE-treated medium for 4 h, followed by 4 h of fresh medium. At the end of the 4 h, culture medium was transferred and centrifuged for 5 min at 2,500 rpm. TNF*α* was analyzed via ELISA (*n* = 4). ^*∗*^ *P* < 0.05: CSE versus CON.

In an effort to elucidate a potential mechanism for conveying the stress from gingiva to muscle, we measured the level of TNF*α* secretion from gingival cells treated with CSE compared with control. We found that prior CSE treatment in gingival cells elicited a highly significant increase in TNF*α* secretion into culture medium that was subsequently used for myotube incubation (Figure 3).

## 4. Discussion

Cigarette smoke exposure bears a significant cardiometabolic burden, increasing the risk of heart disease [19, 23], various pulmonary disorders [24], and metabolic syndrome [7]. However, the mechanism whereby airway smoke exposure elicits a systemic metabolic effect is poorly understood. We have previously found that lung alveolar cells are capable of transmitting an airway signal to systemic tissues like skeletal muscle, altering muscle metabolic function [7]. The results herein are the first to indicate that oral gingival cells may be similarly relevant in the adverse metabolic effects of cigarette smoke exposure.

Our observations of reduced insulin signaling in muscle cells following incubation with conditioned medium from CSE-treated gingival cells carry particular relevance with numerous pathologies associated with smoking. While some smoking-related disorders are unique to the lung, others are systemic and intimately related to disrupted insulin signaling. One of the primary manifestations of insulin resistance is the metabolic syndrome [25–28]. Given the strong causal relationship with cigarette smoke exposure and insulin resistance [7, 15, 17], it is not surprising to note that the relationship with smoke exposure and metabolic syndrome is similarly robust [13, 29].

Further, our findings of disrupted muscle mitochondrial function highlight an additional pathological feature of cigarette smoke exposure. Mitochondria serve an essential role in almost all cells. Previous work has found that cigarette smoke exposure elicits a deleterious effect on mitochondrial physiology, including reduced oxygen use, increased reactive oxygen species (ROS) formation, and compromised ATP production [18, 19, 30]. Our evidence indicating increased ROS formation in muscle cells treated with medium from CSE-treated gingival cells provides compelling evidence of the direct link between smoking and oxidative stress in muscle.

Cigarette smoke is a powerful stimulator of inflammatory pathways throughout the airway, though the effect proximally is more modest than that seen distally or systemically [31–33]. Nevertheless, we found a robust response of gingival cells to produce and secrete TNF*α* in response to CSE. This is relevant; among the myriad proinflammatory cytokines that are increased with smoke exposure, TNF*α* is paramount [34]. TNF*α* is also a likely mediating mechanism that can explain the metabolic disruption in the myotubes incubated with conditioned medium from CSE-treated gingival cells. In addition to inflammation [35], cigarette smoke is known to induce multiple metabolic defects, including mitochondrial dysfunction [36] and reduced insulin resistance [37]. Nonetheless, future studies will further elucidate the role of additional potential mediators, including oxidative stress [3, 38, 39] or ceramides [7, 19, 36, 40, 41].

Ultimately, our findings add evidence to the well established and myriad deleterious consequences of smoke exposure, including pronounced systemic and whole-body metabolic deficits. These efforts may potentially yield a therapy to protect metabolic function in those who are habitually exposed to cigarette smoke or who have trouble quitting.

## References

[1] Jamal A., Agaku I. T., O'Connor E., King B. A., Kenemer J. B., Neff L. (2014). Current cigarette smoking among adults—United States, 2005–2013. *Morbidity and Mortality Weekly Report*.

[2] Gao H., Prasad G. L., Zacharias W. (2014). Combusted but not smokeless tobacco products cause DNA damage in oral cavity cells. *Environmental Toxicology and Pharmacology*.

[3] Reynolds P. R., Kasteler S. D., Cosio M. G., Sturrock A., Huecksteadt T., Hoidal J. R. (2008). RAGE: developmental expression and positive feedback regulation by Egr-1 during cigarette smoke exposure in pulmonary epithelial cells. *The American Journal of Physiology—Lung Cellular and Molecular Physiology*.

[4] Hecht S. S., Carmella S. G., Murphy S. E., Akerkar S., Brunnemann K. D., Hoffmann D. (1993). A tobacco-specific lung carcinogen in the urine of men exposed to cigarette smoke. *The New England Journal of Medicine*.

[5] Azzalini L., Ferrer E., Ramalho L. N. (2010). Cigarette smoking exacerbates nonalcoholic fatty liver disease in obese rats. *Hepatology*.

[6] Fuchs C. S., Colditz G. A., Stampfer M. J. (1996). A prospective study of cigarette smoking and the risk of pancreatic cancer. *Archives of Internal Medicine*.

[7] Thatcher M. O., Tippetts T. S., Nelson M. B. (2014). Ceramides mediate cigarette smoke-induced metabolic disruption in mice. *The American Journal of Physiology—Endocrinology and Metabolism*.

[8] Menke A., Casagrande S., Geiss L., Cowie C. C. (2015). Prevalence of and trends in diabetes among adults in the United States, 1988–2012. *The Journal of the American Medical Association*.

[9] Biddinger S. B., Hernandez-Ono A., Rask-Madsen C. (2008). Hepatic insulin resistance is sufficient to produce dyslipidemia and susceptibility to atherosclerosis. *Cell Metabolism*.

[10] Weyer C., Hanson R. L., Tataranni P. A., Bogardus C., Pratley R. E. (2000). A high fasting plasma insulin concentration predicts type 2 diabetes independent of insulin resistance: evidence for a pathogenic role of relative hyperinsulinemia. *Diabetes*.

[11] Sanyal A. J., Campbell-Sargent C., Mirshahi F. (2001). Nonalcoholic steatohepatitis: association of insulin resistance and mitochondrial abnormalities. *Gastroenterology*.

[12] Strong J. P., Richards M. L. (1976). Cigarette smoking and atherosclerosis in autopsied men. *Atherosclerosis*.

[13] Zhu Y., Zhang M., Hou X. (2011). Cigarette smoking increases risk for incident metabolic syndrome in Chinese men-Shanghai diabetes study. *Biomedical and Environmental Sciences*.

[14] Zein C. O., Unalp A., Colvin R., Liu Y.-C., McCullough A. J., Nonalcoholic Steatohepatitis Clinical Research Network (2011). Smoking and severity of hepatic fibrosis in nonalcoholic fatty liver disease. *Journal of Hepatology*.

[15] Ebersbach-Silva P., Alves T., Fonseca A. T. S., Oliveira M. A. D. N., Machado U. F., Seraphim P. M. (2013). Cigarette smoke exposure severely reduces peripheral insulin sensitivity without changing GLUT4 expression in oxidative muscle of Wistar rats. *Arquivos Brasileiros de Endocrinologia & Metabologia*.

[16] Facchini F. S., Hollenbeck C. B., Jeppesen J., Chen Y.-D. I., Reaven G. M. (1992). Insulin resistance and cigarette smoking. *The Lancet*.

[17] Reaven G. M., Ida Chen Y.-D. (1992). Insulin resistance and cigarette smoking. *The Lancet*.

[18] Gannon A. M., Stämpfli M. R., Foster W. G. (2013). Cigarette smoke exposure elicits increased autophagy and dysregulation of mitochondrial dynamics in murine granulosa cells. *Biology of Reproduction*.

[19] Tippetts T. S., Winden D. R., Swensen A. C. (2014). Cigarette smoke increases cardiomyocyte ceramide accumulation and inhibits mitochondrial respiration. *BMC Cardiovascular Disorders*.

[20] Smith M. E., Tippetts T. S., Brassfield E. S. (2013). Mitochondrial fission mediates ceramide-induced metabolic disruption in skeletal muscle. *Biochemical Journal*.

[21] Pesta D., Gnaiger E. (2012). High-resolution respirometry: OXPHOS protocols for human cells and permeabilized fibers from small biopsies of human muscle. *Methods in Molecular Biology*.

[22] Jheng H.-F., Tsai P.-J., Guo S.-M. (2012). Mitochondrial fission contributes to mitochondrial dysfunction and insulin resistance in skeletal muscle. *Molecular and Cellular Biology*.

[23] Yasue H., Hirai N., Mizuno Y. (2006). Low-grade inflammation, thrombogenicity, and atherogenic lipid profile in cigarette smokers. *Circulation Journal*.

[24] Xu X., Dockery D. W., Ware J. H., Speizer F. E., Ferris B. G. (1992). Effects of cigarette smoking on rate of loss of pulmonary function in adults: a longitudinal assessment. *American Review of Respiratory Disease*.

[25] Romeo G. R., Lee J., Shoelson S. E. (2012). Metabolic syndrome, insulin resistance, and roles of inflammation—mechanisms and therapeutic targets. *Arteriosclerosis, Thrombosis, and Vascular Biology*.

[26] Meshkani R., Adeli K. (2009). Hepatic insulin resistance, metabolic syndrome and cardiovascular disease. *Clinical Biochemistry*.

[27] Bikman B. T. (2012). A role for sphingolipids in the pathophysiology of obesity-induced inflammation. *Cellular and Molecular Life Sciences*.

[28] Reaven G. M. (1993). Role of insulin resistance in human disease (syndrome X): an expanded definition. *Annual Review of Medicine*.

[29] Nakanishi N., Takatorige T., Suzuki K. (2005). Cigarette smoking and the risk of the metabolic syndrome in middle-aged Japanese male office workers. *Industrial Health*.

[30] Knight-Lozano C. A., Young C. G., Burow D. L. (2002). Cigarette smoke exposure and hypercholesterolemia increase mitochondrial damage in cardiovascular tissues. *Circulation*.

[31] van der Vaart H., Postma D. S., Timens W., ten Hacken N. H. T. (2004). Acute effects of cigarette smoke on inflammation and oxidative stress: a review. *Thorax*.

[32] Szulakowski P., Crowther A. J. L., Jiménez L. A. (2006). The effect of smoking on the transcriptional regulation of lung inflammation in patients with chronic obstructive pulmonary disease. *American Journal of Respiratory and Critical Care Medicine*.

[33] Bergström J., Preber H. (1986). The influence of cigarette smoking on the development of experimental gingivitis. *Journal of Periodontal Research*.

[34] Churg A., Dai J., Tai H., Xie C., Wright J. L. (2002). Tumor necrosis factor-*α* is central to acute cigarette smoke-induced inflammation and connective tissue breakdown. *American Journal of Respiratory and Critical Care Medicine*.

[35] Hales C. N., Barker D. J. P. (2001). The thrifty phenotype hypothesis. *British Medical Bulletin*.

[36] Bikman B. T., Summers S. A. (2011). Ceramides as modulators of cellular and whole-body metabolism. *The Journal of Clinical Investigation*.

[37] Hotamisligil G. S., Peraldi P., Budavari A., Ellis R., White M. F., Spiegelman B. M. (1996). IRS-1-mediated inhibition of insulin receptor tyrosine kinase activity in TNF-*α*- and obesity-induced insulin resistance. *Science*.

[38] Khanna A. K., Xu J., Mehra M. R. (2012). Antioxidant N-acetyl cysteine reverses cigarette smoke-induced myocardial infarction by inhibiting inflammation and oxidative stress in a rat model. *Laboratory Investigation*.

[39] Raij L., DeMaster E. G., Jaimes E. A. (2001). Cigarette smoke-induced endothelium dysfunction: role of superoxide anion. *Journal of Hypertension*.

[40] Goldkorn T., Filosto S. (2010). Lung injury and cancer: mechanistic insights into ceramide and EGFR signaling under cigarette smoke. *American Journal of Respiratory Cell and Molecular Biology*.

[41] Nelson M. B., Swensen A. C., Winden D. R., Bodine J. S., Bikman B. T., Reynolds P. R. (2015). Cardiomyocyte mitochondrial respiration is reduced by receptor for advanced glycation end-product signaling in a ceramide-dependent manner. *The American Journal of Physiology—Heart and Circulatory Physiology*.

